# Draft Genome Assembly of *Stutzerimonas* sp. Strain S1 and Achromobacter spanius Strain S4, Two Syringol-Metabolizing Bacteria Isolated from Compost Soil

**DOI:** 10.1128/mra.01150-22

**Published:** 2023-02-23

**Authors:** Daniel P. Brink, Elin M. Larsson, Javier García-Hidalgo, Helga Helgadóttir, Marie F. Gorwa-Grauslund

**Affiliations:** a Division of Applied Microbiology, Department of Chemistry, Lund University, Lund, Sweden; b Division of Biology and Biological Engineering, California Institute of Technology, Pasadena, California, USA; Loyola University Chicago

## Abstract

Two bacterial strains able to use syringol as a sole carbon source were isolated from compost. The isolates, named S1 and S4, were sequenced using the Illumina platform. The final assemblies contained 4.2 Mbp, 63% GC, and 3,912 genes for S1 and 6.2 Mbp, 64% GC, and 5,503 genes for S4.

## ANNOUNCEMENT

Syringol is a major depolymerization product from lignins rich in sinapyl alcohol units (S-units), such as hardwood, cornstalk, and wheat straw lignin (1–3). Compared to other types of lignin units, microbes able to metabolize S-unit-derived aromatics are underrepresented in the literature (4), and among them only a single syringol-utilizing species has been previously reported (5). Here, we present the isolation of two novel bacterial strains capable of growth on syringol as a sole carbon source.

Coarse-grained compost was provided by the Sysav waste company (Malmö, Sweden). Isolation was performed by adding 52.9 g compost to 200 mL NaCl (0.8%, wt/vol), and vortexing vigorously. After settling, 1 mL supernatant was used to inoculate 250-mL shake-flasks with 25 mL M9 medium (6) supplemented with trace elements (7), 2 mM syringol, and 100 μg/mL cycloheximide to prevent fungal growth. After 2 days of incubation at 30°C and 180 rpm, the culture was streaked on agar plates with the same medium. Several sequential restreaks on fresh plates were performed to verify the isolation of pure strains and stability of the syringol-utilization phenotype. Both strains were isolated from the same shake-flask culture.

Genomes from cells cultivated overnight (5 mL M9 medium in conical tubes) were extracted using the GeneJET genomic DNA purification kit (Thermo Fisher, MA, USA). Sequencing was performed at Eurofins Genomics Europe (Germany) using Eurofins’ NGSelect DNA library preparation and the Illumina NovaSeq 6000 S2 PE150 XP platform. The total number of reads was 10,282,622 for strain S1 and 11,937,525 for S4. Read quality and trimming were performed with FastQC v0.11.9 (8) and Trimmomatic v0.39 (9), respectively. Assembly was done with SPAdes v3.15.3 with options: -k 21,33,55,77 --only-assembler --careful (10). An additional SPAdes run with the --plasmid flag (11) was also performed. Assembly metrics were assessed with Quast v4.5.4 (12). Coverage was calculated with BBMap v38.18 (13). Gene prediction and annotation were done with PGAP v6.3 (14, 15). The presence of aromatic funneling pathways was analyzed based on homology with known bacterial proteins (16, 17) using blastp v2.12.0+ (18). Default parameters were used except where otherwise noted.

The assembly of strain S1 resulted in 4.19 Mbp split over 22 contigs, a 63.08% GC content, an *N*_50_ of 428,008 bp, and 686× ± 115× average coverage. The S4 assembly had 6.18 Mbp, 33 contigs, a 64.19% GC content, an *N*_50_ of 370,688 bp, and 541× ± 90× average coverage. No plasmids were successfully assembled. Based on an average nucleotide identity analysis (Table 1), we propose to name the isolates *Stutzerimonas* sp. strain S1 and Achromobacter spanius S4. Totals of 3,912 and 5,503 protein-encoding genes were predicted in the S1 and S4 annotations, respectively. Putative genes from aromatic metabolic pathways were identified in both isolates, including beta-ketoadipate, ferulate, and syringate pathways. Syringol metabolism in bacteria is not well understood, but homologs to a recently described engineered guaiacol-demethylating cytochrome P450 system that enabled conversion of syringol to pyrogallol in Pseudomonas putida (19) were identified in both strains at 24 to 35% sequence identity (locus tags in S1, N8H22_08560 and N8H22_17570; locus tags in S4, N8H69_18620 and N8H69_24105).

**TABLE 1:** Results of the average nucleotide identity analysis used for taxonomic classification of strains S1 and S4^*a*^

Reference assembly (NCBI URL)	ANIb identity (%)	ANIb alignment coverage (%)	ANIm identity (%)	ANIm alignment coverage (%)
Strain S1				
Stutzerimonas degradans FDAARGOS 876 (GCF_016028635.1)	85.64	77.29	87.76	68.59
Pseudomonas phenolilytica RBPA9 (GCF_021432765.1)	85.61	79.97	87.60	70.78
Stutzerimonas frequens TPA3 (GCF_017577085.1)	83.31	72.13	87.50	52.64
Stutzerimonas stutzeri YWX-1 (GCF_019203165.1)	83.21	70.54	87.12	52.78
Stutzerimonas chloritidismutans 6L11 (GCF_020783375.1)	83.05	72.76	87.13	52.99
Stutzerimonas kunmingensis DSM 25974 (GCF_024397575.1)	83.04	70.75	87.17	51.83
Stutzerimonas balearica L1 (GCF_021713615.1)	81.07	67.88	86.06	42.84
[Pseudomonas] *nosocomialis* CCUG 58779 (GCF_005876875.1)	79.90	55.22	85.03	33.20
Stutzerimonas azotifigens DSM 17556 (GCF_000425625.1)	79.76	51.18	84.97	31.95
Stutzerimonas kirkiae P28C (GCF_004327175.1)	79.23	34.83	84.68	20.03
Pseudomonas putida KT2440 (GCF_000007565.2)	76.65	35.06	83.63	12.63
Escherichia coli K-12 substrain MG1655 (GCF_000005845.2)	70.52	6.00	85.33	0.13

Strain S4				
Achromobacter spanius UQ283 (GCF_003994415.1)	98.83*	94.88	98.87*	95.95
*Achromobacter* sp. strain B7 (GCF_003600685.1)	87.35	76.79	88.69	75.19
*Achromobacter* sp. strain ES-001 (GCF_019448355.1)	87.35	77.11	88.67	75.44
Achromobacter piechaudii LMG 1861 (GCF_902860155.1)	86.91	75.12	88.34	71.79
Achromobacter deleyi As-55 (GCF_013116765.2)	84.65	64.95	86.92	57.57
Achromobacter pestifer FDAARGOS 790 (GCF_013267355.1)	84.12	60.38	86.74	51.99
Achromobacter denitrificans FDAARGOS 787 (GCF_013343095.1)	83.87	60.04	86.59	51.40
Achromobacter xylosoxidans A8 (GCF_000165835.1)	83.85	61.41	86.71	52.00
Bordetella petrii DSM 12804 (GCF_000067205.1)	78.99	36.69	84.62	19.84
Burkholderia pyrrocinia LWK2 (GCF_024732225.1)	73.11	12.85	82.20	1.74
Pseudomonas putida KT2440 (GCF_000007565.2)	71.29	7.12	82.01	0.44
Escherichia coli K-12 substrain MG1655 (GCF_000005845.2)	69.60	2.59	81.85	0.03

a Ribosomal 16S sequences were extracted from the assemblies with Barrnap v0.9 (20) and used to select reference genomes from relevant genera to perform average nucleotide identity (ANI) analysis. FastANI v1.33 (21) was used to rapidly query the S1 assembly against the 348 genomes from the *Pseudomonadaceae* family available in the representative RefSeq collection in the NCBI database; in a second stage, S1 was also queried against the 441 genomes from the recently delineated *Stutzerimonas* genus (22). S4 was queried against 734 representative RefSeq *Burkholderiales* genomes. The results from the FastANI screening and BLAST analysis of the 16S sequence were used to decide which genomes to include for the final ANI analysis, which used PyANI v0.2.12 (23) calling on the standard ANI algorithms MUMmer v4.0.0rc1 (24) and BLAST v2.12.0+ (18). To illustrate the taxonomic diversity, only one representative genome from each species was used in the final analysis. Model organisms P. putida and Escherichia coli were included as controls. ANIb, BLAST-based ANI; ANIm, MUMmer-based ANI. A cutoff >95% ANI percent identity was used for species delineation as previously recommended (25). Values that fulfill this criterion are marked with asterisks. The table is sorted by ANIb percent identity in descending order.

### Data availability.

The annotated genome assemblies of strains S1 and S4 have been deposited at DDBJ/ENA/GenBank under the accession numbers JAOEIY000000000 and JAOEJJ000000000, respectively. The versions described in this paper are JAOEIY010000000 and JAOEJJ010000000, respectively. The raw reads for S1 and S4 have been uploaded to the NCBI Sequence Read Archive as SRR21673156 and SRX17674200, respectively. The BioProject numbers for the data sets are PRJNA883164 and PRJNA883223.
